# Therapeutic effects of vitamin D on acetic acid-induced colitis in rats^1^

**DOI:** 10.1590/s0102-865020200040000004

**Published:** 2020-06-12

**Authors:** Refik Bademci, Mümin Alper Erdoğan, Ali Yücel Kara, Gürkan Yiğittürk, Oytun Erbaş

**Affiliations:** I Assistant Professor, Department of General Surgery, Istanbul Medipol University, Istanbul, Turkey. Conception of the study, acquisition of data, manuscript writing, critical revision.; II Assistant Professor, Izmir Katip Çelebi University, Faculty of Medicine, Department of Physiology, Izmir, Turkey. Conception of the study, acquisition of data, methodology, manuscript writing.; III Assistant Professor, Izmir Katip Çelebi University, Faculty of Medicine, Department of Physiology, Izmir, Turkey. Manuscript writing, critical revision.; IV Assistant Professor, Ege University, Faculty of Medicine, Department of Histology and Embryology, Izmir, Turkey. Investigation, manuscript writing, critical revision.; V Associate Professor, Istanbul Bilim University, Department of Physiology, Istanbul, Turkey. Scientific and intellectual content of the study, manuscript writing.

**Keywords:** Calcitriol, Colitis, Tumor Necrosis Factor-alpha, Malondialdehyde, Rats

## Abstract

**Purpose:**

To analyze the effect of calcitriol treatment on acute colitis in an experimental rat model.

**Methods:**

A total of 24 adult Sprague Dawley albino rats were randomly separated into 3 equal groups: control group (n:8), colitis group (n:8), calcitriol administered group (n:8). A single dose of acetic acid (1 ml of 4% solution) was administered intrarectally to induce colitis. Group 1 was given 1 ml/kg 0.9% NaCl intraperitoneally; rats belonging to Group 2 were administered calcitriol 1 µg/kg for 5 days.

**Results:**

Plasma tumor necrosis factor alpha, Pentraxin 3, and malondialdehyde levels were significantly lower in the calcitriol administered colitis group than in the standard colitis group (p<0.01). In the Calcitriol group, there was a significant histological improvement in hyperemia, hemorrhage and necrotic areas in the epithelium compared to the placebo group (p <0.000).

**Conclusion:**

The findings suggest that calcitriol may be an agent that could be used in acute colitis treatment.

## Introduction

Inflammatory bowel disease (IBD) is a condition generally typified by idiopathic, repetitive and often diffuse inflammation of the colon and the rectum mucosa. Ulcerative colitis (UC) and Crohn’s disease (CD) are the major subtypes of IBD. Though the etiology of IBD is not yet fully comprehended, many genetic, environmental and immunological factors play a role and it is thought that intestinal microflora, mucosal immunoreaction, autoimmune reactions and especially oxidative stress, play a role in the pathophysiology of IBD^1,2^. Many inflammatory factors such as TNF-α, malondialdehyde (MDA), and pentraxin-3, increase in levels parallel with an increase in IBD inflammation, and thus histological evidence of inflammation in tissues can be observed^3^.

The role of reactive oxygen species (ROS) in the pathogenesis of inflammatory bowel disease (IBD) has been stressed in recent years^2^. Excessive inflammation and oxidative stress have pivotal roles in ulcerative colitis disease pathogenesis^3^. Inflammatory mediators are known to be secreted from migrated granulocytes in the inflamed mucosa in this disease^2^. Evidence suggests that the cascade of free radical production and subsequent lipid peroxidation reduce the cellular antioxidant capacity, resulting in colonic inflammation.

Recently, increased oxidative stress and decreased antioxidant defense system have been shown by mucosal biopsies in IBD^4^. The efficacy of the current standard therapy, e.g., aminosalicylates, was found to be related to their antioxidant and scavenging action^4^. Inhibition of lipid peroxidation or scavenging of oxygen free radicals would provide an important protective and therapeutic treatment for ulcerative colitis^5^. Additionally, the effects of various antioxidant substances in IBD were investigated as potential therapeutic agents^6,7^. Thus we preferred vitamin D, the antioxidant effects of which were demonstrated previously, in the treatment of experimental acute colitis in this study.

Furthermore, secreted proinflammatory factors are likely pathological factors and weaken the mucosal barrier function and intestinal permeability. Previous studies of IBD have shown reduced levels of conjugated complex proteins in the intestinal mucosa^4,8^. The deteriorated intestinal epithelial barrier function may increase intestinal inflammation by causing permanent immune reactions^9^. Literature studies have shown that a wide variety of agents in the treatment of colitis such as ozone, dexpanthenol, nesfatin-1, ilioprost, and anti-TNF have been tried and demonstrated in experimental studies^1,9^. The basis of the treatment depends on inflammatory mediator secretion, chemotaxis inhibition and antioxidant treatment, so it is important to identify inflammation-reducing agents for the treatment of IBD.

The histopathological appearance of human IBD can be reproduced with experimental colitis induced by acetic acid in laboratory animals. The overproduction of oxidative mediators, which are known to be significant in the pathophysiology of colitis, is caused by the sensitivity of colonic tissue to acetic acid^6,10^.

Aminosalicylates are currently used as standard therapy and the efficacy of this treatment has been shown to be associated with the antioxidant and scavenging action^11^. It has been suggested that inhibiting lipid peroxidation or scavenging of oxygen free radicals could lead to significantly protective remedy for UC^4^. The effects of various antioxidants have also been evaluated as potential therapeutic agents for IBD^6,7^. Therefore, vitamin D was selected for use in this study of the treatment of experimental acute colitis as its antioxidant effects have already been previously demonstrated.

D vitamins are the most prominent effectors of bone metabolism, although they have also been implicated in several chronic diseases such as autoimmune and infectious diseases, diabetes, hypertension and cancer^11^. While the number of IBD cases around the equator region has been reported to be very low, a greater number of IBD cases have been observed in the northern parts of the world, which suggests that vitamin D deficiency may cause IBD^12^. Many experimental colitis studies have shown that colonic antimicrobial activity is dysregulated in vitamin D deficiency^13,14^. Vitamin D Receptor (VDR) is furthermore supplemented by many immune-system cells. Experimental studies by Liu et al. showed that the number of global vitamin D receptors in mice with colitis was reduced and that VDR has a protective effect on mucosal epithelium^15^. In addition, one of the tasks of vitamin D is to regulate the T cell response, which is also important in body defense. Through both direct and indirect pathways, D vitamins repress pathogenic Th1 and Th17 cell responses, regulating human natural killer, Treg cells and CD8α, and suppressing inflammation in the gastrointestinal tract^4,16^. Many previous reports have also shown that vitamin D deficiency is a potential environmental cause of IBD^17-22^.

Oxidative stress is defined as a significant imbalance between reactive oxygen species (ROS) production and antioxidant defenses. It induces the modifications in signaling pathways and potential tissue damage. There is several evidence supporting the antioxidant activity of vit D3 (cholecalciferol) in the oxidative stress diabetes. The results in some experimental studies implied that vit D3 administration in diabetic mice helps to diminish the ROS formation by the suppression of the gene expression of NADPH oxidase^18,19^. According to the literature, calcitriol could enhance the pathway of ROS removal, by increasing the intracellular pool of reduced GSH, partially through upstream regulation of glutamate-cysteine ligase (GCL) and glutathione reductase (GR) genes expression^19^. GCL is a key enzyme that is involved in synthesis of GSH^18^. With its high antioxidant capacity and anti-inflammatory activity, vitamin D would be expected to reduce injury and/or improve tissue after injury from ulcerative colitis.

The objective of this research was to evaluate and analyze the effects of calcitriol on acute colitis in a rat model of acute colitis induced by acetic acid.

## Methods

### *Animals*

Experimental manipulations and surgical procedures were carried out in the Ege University, Faculty of Medicine, Animal Research Laboratory.

The study has used 24 mature Sprague Dawley male albino rats, each with an approximate weight of 0.2 to 0.22 kg (220 grams to 220 grams). The animals were provided with food and water and were stored in pairs in stainless steel cages at a mean temperature of 22 ± 2°C and a 12-h light/dark cycle. All the procedures used in this experimental study were authorized by the Animal Research Ethics Committee. The experiment was conducted in accordance with the Guide for the Care and Use of Laboratory Animals, as authorized by the guidelines set forward by the National Institutes of Health (USA).

### *Experimental protocol*

Acute colitis was induced in 16 rats through rectal administration of a 4% solution of acetic acid (AA) in a volume of 1 ml. AA was not administered to the control group (n=8).

Ether anesthesia was administered and then acetic acid (AA) was administered via a 6 cm soft catheter inserted in the anus. While the catheter was still in place, 1 ml of air was applied to ensure the complete spread of AA throughout the colon. Care was taken to prevent leakage from the anus. Then, the 16 rats with induced colitis, were randomly divided into 2 groups. Group 1 (placebo group, 8 rats) was given 1 ml/kg 0.9% NaCl intraperitoneally (i.p.); Group 2 (calcitriol group, 8 rats) was given calcitriol (Calcijex 1 microg/ml, Abbott) 1 µg/kg for 5 days. After euthanasia of the animals, blood samples for biochemical analysis were taken via cardiac puncture. The rectum and colon were removed for histopathological and biochemical examination.

### *Macroscopic evaluation*

Abdominal entry was made to expose the colon, which was then excised starting from the point closest to the rectum and continuing to the splenic flexure level. Longitudinal dissection was made along the mesenteric border. The mucosa was washed in saline solution, then the samples were scored macroscopically from examination using a simple magnifying glass. The scoring system used was: 0 = no damage; 1 = patch type superficial hyperemia; 2 = generalized patch type hyperemic regions with normal mucosa in between; 3 = generalized hyperemia and hemorrhage. Following the macroscopic analysis, tissue samples of full thickness were taken from the distal parts of the colon adjacent to the rectum.

### *Histopathological evaluation*

Haematoxylin and eosin staining was applied to formalin-fixed rectum and colon sections (4 μm), which were then photographed using an Olympus C-5050 digital camera installed on an Olympus BX51 microscope.

Scoring was applied according to the MacPherson and Pfeiffer criteria (1976): 0 = intact epithelium, no leukocytes or hemorrhage: 1 = <25% disrupted epithelium, focal leukocyte infiltrates, and focal hemorrhage; 2 = 25% disrupted epithelium, focal leukocyte infiltrates, and focal hemorrhage; 3 = 50% disrupted epithelium, widespread leukocytes, and hemorrhage;

### *Measurement of plasma TNF-α levels*

Levels of plasma TNF-α were measured using the enzyme-linked immunosorbent assay (ELISA) commercial kit (Biosciences). In accordance with the kit protocol, plasma samples were diluted 1: 2 and TNF- α was determined in duplicate at a detection range of <2 pg/ml.

### *TNF- α in the rectum*

Using a glass homogenizer, the frozen rectum tissue samples were homogenized in 1 ml of buffer, comprising 1 mmol/L PMSF, 1 mg/L pepstatin A, 1 mg/L aprotinin, and 1 mg/L leupeptin in PBS solution (pH 7.2). Following centrifugation at 12,000 rpm for 20 minutes at 4°C, the supernatant was collected, and the total protein was determined using the Bradford method. The TNF levels in the tissue supernatants were measured using a rat TNF-α-specific ELISA kit (eBioscience, Inc, San Diego, CA, USA). TNF-α measurements were taken in a step-by-step fashion, as per the kit protocol. In accordance with the manufacturer’s speciﬁcations, the inter-assay and intra-assay coefﬁcients of variation for TNF-α were 7.9%–8.2% and 6.1–6.5%, respectively. The TNF-α minimum level detected for this assay was 30pg/ml. The cytokine amount present in brain tissue was depicted as pg/mg tissue.

### *Analysis of the plasma pentraxin-3 level*

Measurement of the plasma pentraxin-3 (PTX3) levels was conducted with 100 μl sample using a PTX3 kit (USCN Life Science Inc, Wuhan, China) and standard ELISA apparatus at 450 nm. In accordance with the kit protocol, PTX3 levels were determined in duplicate. PTX3 setup had a detection range of 0.078- 5 ng/ml.

### *Measurement of lipid peroxidation*

For the determination of lipid peroxidation in plasma samples, malondialdehyde (MDA) levels were measured as thiobarbituric acid reactive substances (TBARS). After the addition of trichloroacetic acid and TBARS reagent to the plasma samples, these were then mixed and incubated at 100°C for 60 min. Following a cooling period on ice, the samples then underwent centrifuging at 3000 rpm for 20 min and supernatant absorbance was measured at 535 nm. MDA levels were expressed as nM and tetraethoxypropane was used for calibration.

### *Statistical analysis*

Data obtained in the study were analyzed statistically utilizing SPSS version 15.0 for Windows operating system. Comparisons of groups of parametric variables were made using the Student’s t test and analysis of variance (ANOVA). Comparisons of non-parametric variables were made using the Mann Whitney U test. Results were stated as mean ± standard error of mean (SEM). A value of p < 0.05 was accepted as statistically significant and p < 0.001 as statistically highly significant.

## Results

**Figure 1:**
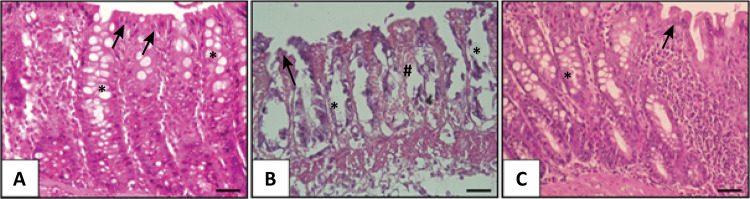
Hematoxylin and eosin stain. a) Control Group Rectum normal epithelium (*arrow*) and gland (*). b) Colitis + 0.9% NaCl administered group, disrupted epithelium, gland and hemorrhage (#). c) Colitis + 1 µg/kg calcitriol administered group, healed epithelium and gland. (x40 magnification)

When the control group, placebo group and the Vitamin D group were compared in a histopathological manner, the colitis group showed a higher ratio of hyperemia: hemorrhage and necrotic areas in the epithelium (p <0.01) compared to the placebo group and the vitamin D group (p <0.01). In the group receiving vitamin D, hyperemia, hemorrhage and necrotic areas showed a significant improvement (p <0.000) (Table 1, Fig. 1).

**Table 1:** Histopathological and macroscopic scores of the groups.

	Control Group	Colitis + 0.9% NaCl administered group	Colitis + 1µg/kg calcitriol administered group
Macroscopic score	0.5 ± 0.18	2.6 ± 0.18 *	1.5 ± 0.27 **
Histopathological score	0.25 ± 0.16	3.5 ± 0.27 *	2.3 ± 0.31 **

* p<0.000, Control group compared with Colitis +0.9% NaCl administered group

** p<0.01, Colitis + 1 µg/kg calcitriol administered group compared with Colitis + 0.9% NaCl administered group

It was observed that the plasma TNF-α level had significantly increased in the colitis group compared to the control group (p <0.01) and the level of TNF-α from the rectal tissue was significantly increased in the colitis group (p <0.000). In the vitamin D group, the TNF-α level was found to be close to that of the control group in both plasma and rectum tissues (p <0.05) (Table 2).

**Table 2:** Plasma TNF-alpha, Plasma MDA, Rectum TNF-alpha, Plasma Pentraxin-3 levels of the groups.

	Control Group	Colitis + 0.9% NaCl administered group	Colitis + 1 µg/kg calcitriol administered group
Plasma TNF-alpha (pg/ml)	27.3 ± 4.1	49.7 ± 4.9 *	27.8 ± 3.9 **
Plasma MDA (nM)	67.3 ± 3.7	123.5 ± 3.6 #	84.9 ± 8.6 **
Rectum TNF-alpha (pg/mg tissue)	112.6 ± 5.9	157.5 ± 5.4 ҂	119.9 ± 5.7 **
Plasma Pentraxin-3 (ng/ml)	1.2 ± 0.16	2.8 ± 0.4 ҂	1.8 ± 0.23 **

* p<0.01, Colitis + 0.9% NaCl administered group compared with Control group

҂ p<0.000, Colitis + 0.9% NaCl administered group compared with Control group

# p<0.000, Colitis + 0.9% NaCl administered group compared with Control group

** p<0.05, Colitis + 1 µg/kg calcitriol administered group compared with Colitis + 0.9% NaCl administered group

Plasma MDA levels were significantly higher in the colitis group and were decreased in the Vitamin D group (p <0.05). The plasma pentraxin level was found to be high in the colitis group (p <0.000) and decreased in the Vitamin D group (p <0.05) (Table 2).

## Discussion:

The chronic condition of IBD lowers the life quality for the many of the people who have been affected. It usually manifests with destruction of the colon mucosa and consequent diarrhea attacks. Surgical treatments may be curative, as extensive resections reduce the life quality and medical treatments are generally preferred for cases with limited, mild and moderate inflammation^16^. Acute colitis hemorrhage is a condition of high mortality and morbidity for surgeons, which can lead to life-threatening complications ranging from perforation to toxic megacolon^20^. Therefore, acute colitis conditions require medical agents to stabilize the patients. It has been shown in some experimental colitis studies that vitamin D is useful for healthy bowel operation^22^, and that the loss or overexpression of VDR expression, worsens or alleviates symptoms in experimental colitis models, respectively, and it is seen as a potential cause of increased mucosal permeability, increased intestinal epithelial cell apoptosis, increased mucosal bacterial burden, and increased colitis symptoms in autophagy^22,23^. In many epidemiological studies, it is stated that there is a relationship between vitamin D3 levels and the life quality of IBD patients^22^. It has been observed that supplementing vitamin D in patients with active UC with low level of vitamin D decreases the clinical activity of the disease, decreases the levels of inflammation markers and improves histopathological alterations^24^. Vitamin D plays a role in cell proliferation and differentiation, as well as immunomodulation^25,26^. The vitamin D supplementation in the treatment of some immune-related diseases is increasing. However, there is not sufficient information on the efficacy of its use in acute colitis treatment. In this study, our objective was to determine the efficacy of biochemical and histological changes in rats by giving vitamin D in acute colitis model, which is induced by acetic acid in a rat model.

In colonic wall injury, proinflammatory reaction is associated with increased mucosal concentration of interleukin 1β (IL-1β) and TNF-α. While IL-1β directly affects the proinflammatory cascade, it also stimulates the expression of proinflammatory prostaglandins, primarily IL-6 and TNF-α. In ulcerative colitis, a response to treatment may be obtained with TNF-α antagonist^22^. Patients with UC may respond to treatment with TNF-α antagonists^23^. The TNF-alpha blocker Infliximab is known to improve the course and prognosis of acute severe UC and reduce colectomy rates^27^. Vitamin D has been reported to suppress TNF-α production^24,25^. A single dose of vitamin D3 injection in UC patients was found to significantly reduce serum TNF-α, IFN-γ, and IL12p70 levels^28^. Various studies related to rats (specifically Muridae family of rats) have demonstrated a conclusive biological relationship between vitamin D levels and the development of colitis. It has been demonstrated in rats which have been induced with IBD, that gastrointestinal mucosal inflammation is accompanied by an increase in the levels of proinflammatory cytokines (eg, interleukin 2 [IL-2], IL-12, interferon γ, TNF-α)^24-26^. Similarly, vitamin D–deficient mice often manifest diarrhea, wasting disease, and subsequent death, while mice having sufficient levels (or more) of Vitamin D may not necessarily develop any Inflammatory Bowel Disease symptoms. Administration of exogenous vitamin D or a VDR agonist in IBD mouse models has been shown to reduce TNF-α and suppress colitis. In addition, this study has demonstrated in the experimental IBD model that there was a TNF alpha elevation and that they were released from TNF alpha monocytes and TNF-α macrophages and that this would cause an increase in the free radical production and inflammation. Free radicals can play a role in the etiology of IBD^21,22^. In the current study, it was observed that the acute colitis model increased the level of TNF-α, while vitamin D application decreased the level of TNF-α.

Pentraxin-3 is produced from regional macrophages, dendritic cells, fibroblasts, smooth muscle cells, adipocytes, and vascular endothelial cell neutrophils in response to primary inflammatory signals such as TNF-α and IL-1β^28,29^. Pentraxin-3, a member of the pentraxin family, contributes to the progression of the immunoreaction and acute inflammation. Normally at very low levels, the release and production of PTX3 is increased in inflammation or damage to the body and is thought to be more effective in the identification of inflammatory disease than CRP^30,31^. The amount of PTX3 that responds as an acute phase reactant is proportional to the severity of the damage^18^. It has also been shown to be elevated in chronic diseases such as atherosclerosis, obstructive pulmonary diseases, allergic asthma, vasculitis, lupus, and rheumatoid arthritis^30,31^. Previous studies have also indicated that plasma pentraxin levels in active colitis patients increase in a similar manner to that observed in the current study^32^. Serum PTX3 levels have been found significantly increased in patients with active CD as compared to patients in remission. It was stated that the level of PTX3 can be used as a novel biomarker because it reflects the activity of the disease^33^. In the present study, administration of vitamin D statistically lowered PTX3 levels when compared to the placebo group.

As the end product of lipid peroxidation, MDA reflects the level of lipid peroxidation in the tissue. The increase in MDA level is due to tissue damage, lipid peroxidation and the severity of the inflammation ^33^. On the other hand, it is seen that lipid peroxidation is a crucial factor for cellular injuries in IBD. Reactive radicals attack unsaturated fatty acids in lipids in order to initiate a free radical chain reaction as well as to cause a disturbance of the membrane function^7^. In a previous acetic acid-induced colitis model, the occurrence of oxidative stress was seen to lead to a significant increase in the MDA level^2^. In the current study, the MDA level detected in the acute colitis model with Vitamin D administered was lower than that of the placebo group.

Macroscopic and histopathological evaluation of the existing inflammatory response in the colon is the diagnostic gold standard^7^. Findings such as ulcer, coagulative necrosis of the mucosa associated with hemorrhage and inflammation in the epithelium, a decrease in goblet cells, death of epithelial cells, and damage to intestinal crypts have been shown in a colonic intestinal model^34^. In the current study, hemorrhage and necrotic areas of mucosa in the group given vitamin D were found to be less than those of the placebo group. These findings are in agreement with those of previous similar studies^34,35^.

In addition, our study established that vitamin D decreased the production of PTX3, MDA and TNF-α in the colonic tissue. Thus, vitamin D produced a potent anti-inflammatory effect by scavenging radicals and eliminating cytokines causing colonic inflammation in this model.

## Conclusions

Our results showed that vitamin D inhibited not only oxidant damage but also inflammatory cytokines and histologically improved the colonic inflammation induced by AA in rats. This study suggests that vitamin D is an effective anti-inflammatory and antioxidant and that it may be a promising therapeutic option for ulcerative colitis. However, there is a need for more detailed studies in order to assess the possible relationships between colitis induced with acetic acid and vitamin D.

## Footnotes

1 Research performed at Ege University, Faculty of Medicine, Animal Research Laboratory, Izmir, Turkey.
